# Rapid Biodiversity Assessment and Monitoring Method for Highly Diverse Benthic Communities: A Case Study of Mediterranean Coralligenous Outcrops

**DOI:** 10.1371/journal.pone.0027103

**Published:** 2011-11-02

**Authors:** Silvija Kipson, Maïa Fourt, Núria Teixidó, Emma Cebrian, Edgar Casas, Enric Ballesteros, Mikel Zabala, Joaquim Garrabou

**Affiliations:** 1 Institut de Ciències del Mar (ICM-CSIC), Barcelona, Catalonia, Spain; 2 UMR 6540 - DIMAR CNRS - Université de la Méditerranée, Centre d'Océanologie de Marseille, Station Marine d'Endoume, Marseille, France; 3 Division of Biology, Faculty of Science, University of Zagreb, Zagreb, Croatia; 4 Departament de Ciències Ambientals, Facultat de Ciències, Universitat de Girona, Girona, Catalonia, Spain; 5 Centre d'Estudis Avançats de Blanes (CEAB-CSIC), Blanes, Catalonia, Spain; 6 Departament d'Ecologia, Universitat de Barcelona, Barcelona, Catalonia, Spain; National Institute of Water & Atmospheric Research, New Zealand

## Abstract

Increasing anthropogenic pressures urge enhanced knowledge and understanding of the current state of marine biodiversity. This baseline information is pivotal to explore present trends, detect future modifications and propose adequate management actions for marine ecosystems. Coralligenous outcrops are a highly diverse and structurally complex deep-water habitat faced with major threats in the Mediterranean Sea. Despite its ecological, aesthetic and economic value, coralligenous biodiversity patterns are still poorly understood. There is currently no single sampling method that has been demonstrated to be sufficiently representative to ensure adequate community assessment and monitoring in this habitat. Therefore, we propose a rapid non-destructive protocol for biodiversity assessment and monitoring of coralligenous outcrops providing good estimates of its structure and species composition, based on photographic sampling and the determination of presence/absence of macrobenthic species. We used an extensive photographic survey, covering several spatial scales (100s of m to 100s of km) within the NW Mediterranean and including 2 different coralligenous assemblages: *Paramuricea clavata* (*PCA*) and *Corallium rubrum* assemblage (*CRA*). This approach allowed us to determine the minimal sampling area for each assemblage (5000 cm^2^ for PCA and 2500 cm^2^ for CRA). In addition, we conclude that 3 replicates provide an optimal sampling effort in order to maximize the species number and to assess the main biodiversity patterns of studied assemblages in variability studies requiring replicates. We contend that the proposed sampling approach provides a valuable tool for management and conservation planning, monitoring and research programs focused on coralligenous outcrops, potentially also applicable in other benthic ecosystems.

## Introduction

Coastal ecosystems are among the most diverse, highly productive and complex biological systems [1]. At the same time, they are highly threatened by a combination of anthropogenic impacts, such as overfishing, habitat loss, eutrophication, introductions of exotic species and climate change [2], [3], leading to profound structural and functional changes [4], [5]. However, future shifts in the species composition of assemblages cannot be evaluated without knowledge and understanding of the present state of marine biodiversity. Obtaining this baseline information represents a key step in exploring future modifications of coastal ecosystems.

The Mediterranean Sea is considered a marine biodiversity hotspot, harboring approximately 10% of world's marine species while occupying only 0.82% of the ocean surface [6], [7]. Unfortunately, the impacts of human activities are proportionally stronger in the Mediterranean than in the other seas, raising concerns regarding threats to the conservation of the rich Mediterranean biodiversity [6]. Coralligenous outcrops, which are hard bottoms of biogenic origin that thrive under dim light conditions, are among the habitats faced with major threats in the Mediterranean Sea. These outcrops are highly diverse (harboring approximately 20% of Mediterranean species) and exhibit great structural complexity [8]–[10]. The species that dominate coralligenous seascapes are encrusting calcareous algae, sponges, cnidarians, bryozoans and tunicates. Some of the engineering species in these environments are long-lived, and their low dynamics make coralligenous outcrops exceptionally vulnerable when faced with sources of strong disturbances, such as destructive fishing practices, pollution, invasive species or mass mortality outbreaks [8], [11]–[13].

The immediate consequences and long-lasting effects of these disturbances have mostly been addressed at the population level, focusing on certain structurally important species (e.g., [12], [14]–[18]). Despite the ecological, aesthetic and economic value of coralligenous outcrops, coralligenous biodiversity patterns at the community level over regional scales remain poorly understood ([8], [19] and references therein). This lack of information is partially due to the complexity involved in studying these highly diverse systems with slow dynamics, coupled with general logistical constraints related to sampling at deep rocky habitats.

Most of the previous studies at the assemblage level have been largely descriptive [20]–[23]. There are a few quantitative studies available, restricted to small or medium spatial scales, but their results are not comparable due to the differences in sampling methodology (e.g., scraped samples *vs.* photographic sampling) [10], [24]–[28]. Therefore, an accurate overview of the general biodiversity patterns associated with coralligenous outcrops is lacking.

Ecologists, conservation practitioners, managers and policy makers highlight the need to develop cost-effective sampling methods to provide comparative measures of biodiversity and to create a platform of “biodiversity baselines”. There is currently no single sampling method that has been demonstrated to be sufficiently representative to provide adequate community assessment and monitoring in coralligenous outcrops [29].

To ensure the representativeness and time- and cost-efficiency of any benthic community survey, aiming to capture the original community structure and to account for its natural variability, an adequate sampling unit size and sampling effort (i.e. the number of replicates) should be determined [30], [31]. Therefore, when the goal is to assess the complexity of the system, a good representation of the species pool should be achieved and therefore the minimal sampling area for the assemblage should be defined, i.e. the sampling unit size over which an increase of area does not yield a significant increase in the number of species [32]–[34]. Both the sampling unit size and sampling effort will influence the representativeness of a sample data set in terms of accuracy (the ability to determine the true value) and precision (the ability to detect differences) of the estimates [29]. While accuracy and precision generally increase with sampling effort [29], the high small-scale heterogeneity of coralligenous habitats additionally implies that large sampling areas are required to achieve representative results [8]. However, optimization of the sampling strategy is indispensable given the considerable depths where coralligenous outcrops usually develop and the limited information that can be obtained in the restricted diving time.

Taking into account the priorities and activities defined by the Action Plan for the Conservation of the Coralligenous [13], we aimed to provide guidelines for the application of a rapid, non-destructive protocol for biodiversity assessment and monitoring in coralligenous habitat. The sampling procedure used in this study was designed to assess the natural spatio-temporal variability of coralligenous outcrops, which is crucial information for *a posteriori* assessment of the impact of anthropogenic activities.

The aims of this study were three-fold: (1) to determine the minimal sampling area required to assess the sessile macrobenthic species composition in the studied assemblages, (2) to estimate the minimal sampling effort needed to obtain a good representation of the number of species and the complexity of the overall community and (3) to explore the capacity of the proposed approach to account for assemblage composition variability on different spatial scales and among different assemblages. The application of this approach to characterizing coralligenous outcrops and detecting future changes was also assessed.

## Materials and Methods

### Ethics Statement

Institut de Ciències del Mar (ICM-CSIC), Centre d'Océanologie de Marseille, University of Zagreb (Faculty of Science), Universitat de Girona (Facultat de Ciències), Centre d'Estudis Avançats de Blanes-CSIC and Universitat de Barcelona approved this study.

### Communities studied and study areas

**Figure 1:**
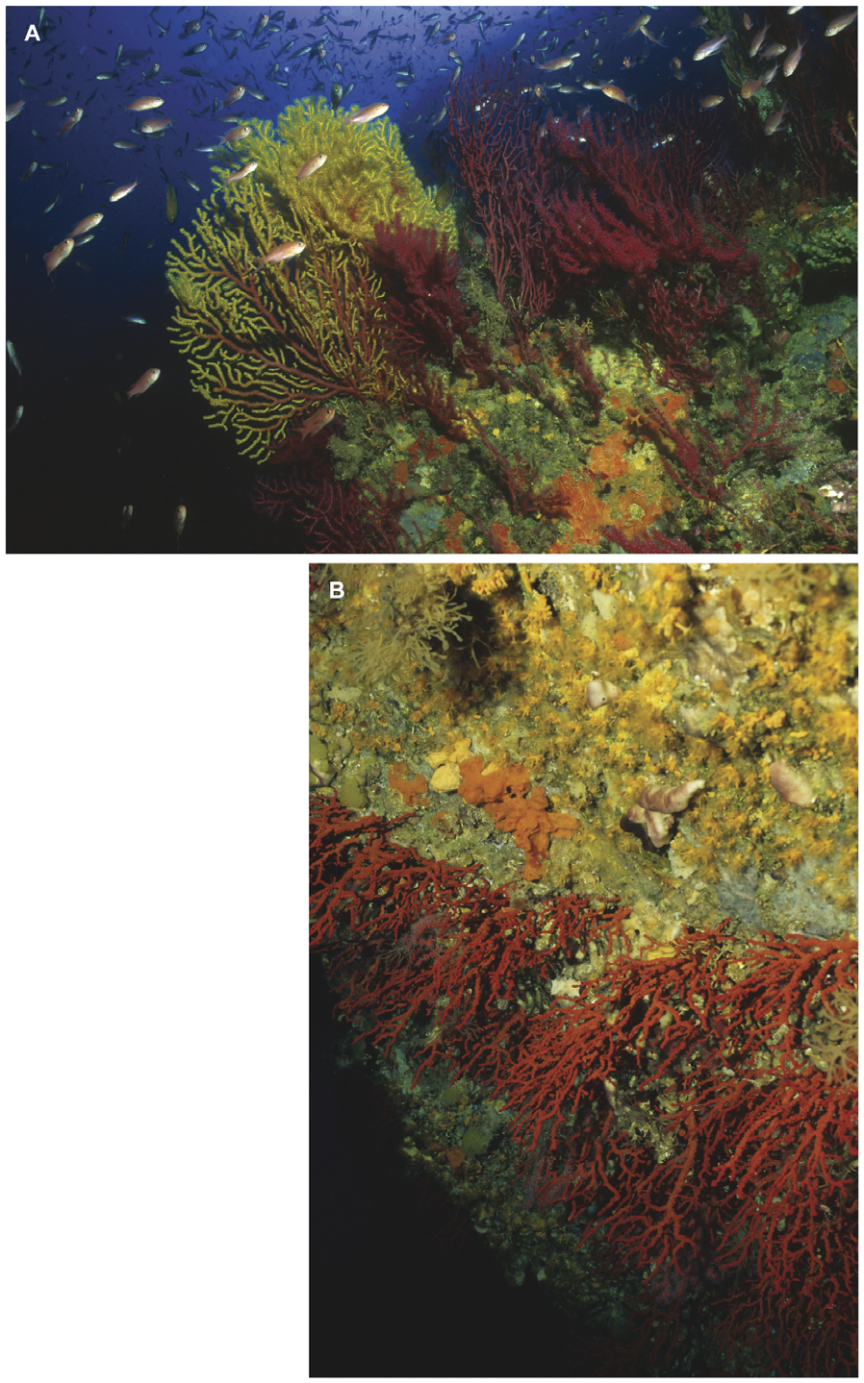
General aspect of 2 facies of the coralligenous outcrops considered in this study. (A) *Paramuricea clavata* assemblage (*PCA*) and (B) *Corallium rubrum* assemblage (*CRA*). Photos by E. Ballesteros.

Coralligenous outcrops comprise a complex of assemblages ranging from algal dominated ones to others completely dominated by macroinvertebrates with almost no algal growth [8]. Here we selected two assemblages that are dominated by the long-lived gorgonians *Paramuricea clavata* (Risso 1826) and *Corallium rubrum* (L. 1758) (Fig. 1) and that displayed the same aspect at all studied sites, always thriving under dim light conditions. The *P. clavata* assemblage (hereafter *PCA*) was sampled on rocky walls at depths ranging from 17 to 24 m, whereas the *C. rubrum* assemblage (hereafter *CRA*) was sampled on overhangs and cave entrances at depths between 14 and 20 m. Further, we consider these assemblages among the most complex ones within the coralligenous outcrops, enabling us to develop a representative sampling method that would perform well in less complex coralligenous assemblages.

**Figure 2:**
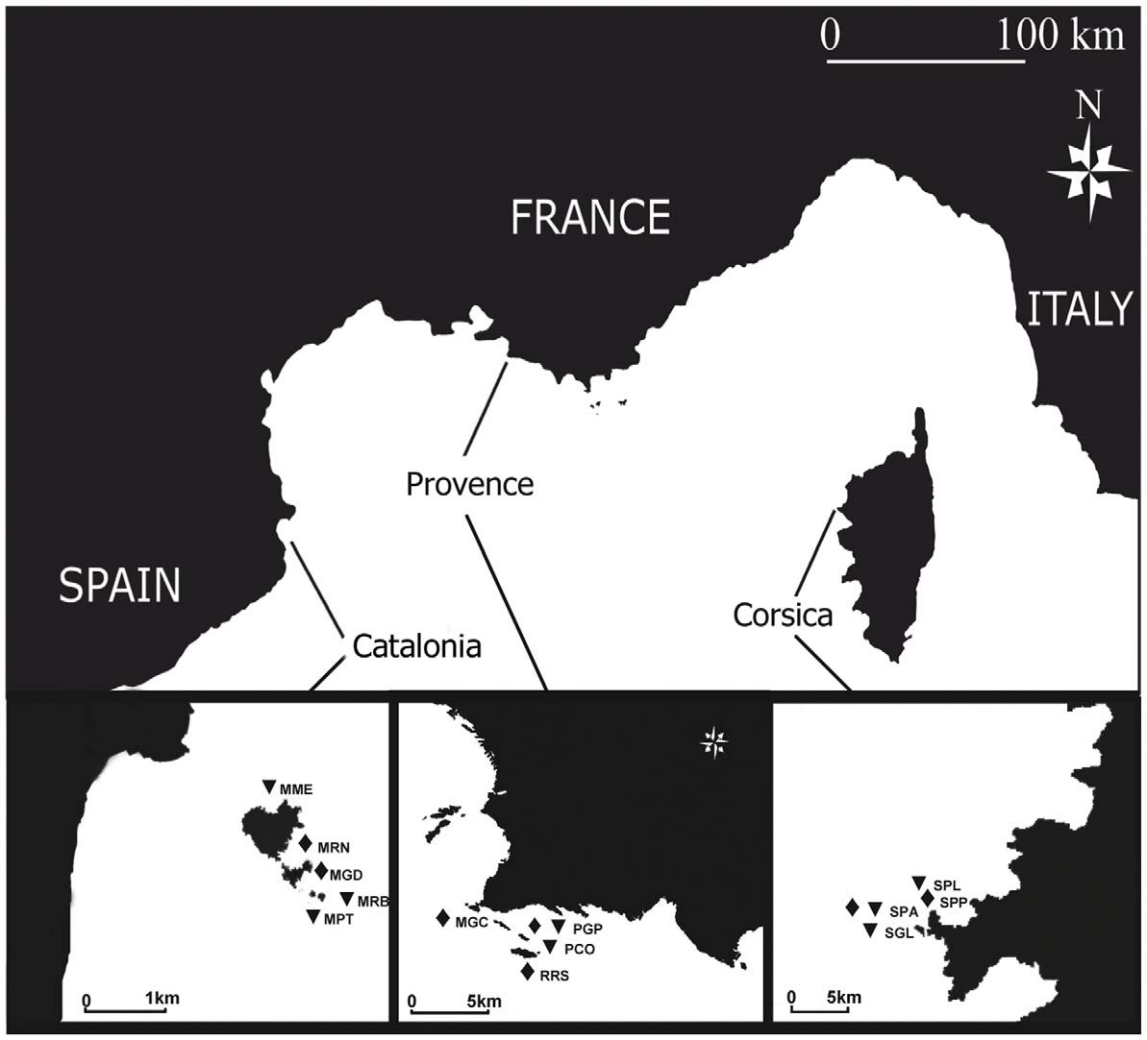
Map of the study area in the NW Mediterranean Sea. Three studied regions in the NW Mediterranean and sites within them (triangles  =  sites with *Paramuricea clavata* assemblage and diamonds  =  sites with *Corallium rubrum* assemblage). See Table 1 for site abbreviations.

We studied a total of 15 sites (8 sites for *PCA* and 7 sites for *CRA*) located in three regions: northern Catalonia, Provence and Corsica, covering more than 400 km of the coastline (Fig. 2). Two to three sites per region and assemblage were sampled (sites within regions were separated by hundreds of meters to a few kilometers). The selected regions encompass a high temperature-productivity gradient in the NW Mediterranean. Provence is characterized by cold, relatively eutrophic waters maintained by local upwellings. Northern Catalonia is characterized by waters largely influenced by river discharges [35], [36], whereas Corsica is characterized by warmer and more oligotrophic waters [36]. Therefore, each region presents particular environmental conditions, thus providing a good dataset for testing the potential of the biodiversity assessment method for detecting natural inter-regional variability. In fact, along this gradient, shifts in the zonation patterns have been reported with coralligenous assemblages developing at shallower depths in the cold-eutrophic areas than in the warm-oligotrophic ones [37]. The observed depth of the coralligenous outcrops ranges from 10 to 50–55 m in Provence (Marseille area) and Catalonia (Medes Islands) [38]–[40] while in Corsica it ranges from 20 to 80 m [38].

### Photographic sampling

The proposed method for biodiversity assessment was based on analysis of the presence/absence of macro-species dwelling in the understory of the selected assemblages that were identified from photographs (see below). To facilitate identification of these species, we sampled the assemblages using quadrats of 25×25 cm for *PCA* and 20×20 cm for *CRA*. The photographs were taken with a Nikon D70S digital SLR camera fitted with a Nikkor 20 mm DX lens and housed in Subal D70S housing. Lighting was provided by two electronic strobes fitted with diffusers. Sampling was conducted during spring and summer of 2006 and 2007. A total of 475 and 486 photographs were analyzed for *PCA* and *CRA*, respectively.

### Species identification

Using these photographs, species were identified to the lowest possible taxonomic level. When further clarification was needed, working with marked plots (see below) allowed us to precisely track down an organism in the field and collect a voucher specimen. Thus, a total of 208 specimens were collected for further identification in the laboratory. Visually similar taxa that could not be consistently identified from photographs were grouped as indicated in Table S1. Furthermore, because the time of sampling differed for different sites, the species showing clear seasonality were excluded from the subsequent analysis (see Table S1).

### Determination of a sampling method for biodiversity assessment in coralligenous outcrops

To determine the sampling method to be used for biodiversity assessment in coralligenous outcrops, we established the minimal sampling area (hereafter MSA) and minimal sampling effort required to provide good estimates of the species number and composition for each studied assemblage.

#### a) Estimation of minimal sampling areas

To estimate MSA, we analyzed the species-area relationship [32], [33], [41], [42], taking into account the spatial arrangement of species, to obtain a good representation of the species pool, as well as the structure of the community [34], [43].

Therefore, we applied a spatially explicit design based on contiguous sampling of quadrats arrayed to cover rectangular plots. At each site, we employed plots ranging from 3.2 to 4 m^2^ for *PCA* and from 1.76 to 3.72 m^2^ for *CRA*. The plots were marked with screws fixed to the rock by putty, and quadrats inside the plots were sequentially positioned and photographed. Overall, 51 to 64 quadrats were photographed per site for *PCA*, whereas 44 to 93 quadrats were photographed per site for *CRA.*

For further determination of MSA, we followed the method described by Ballesteros [44]. A species-area curve for each plot was produced from the subset of all possible combinations of increasing numbers of the originally ordered contiguous quadrats. Thus, mean values of species numbers for successively larger areas were obtained and plotted *vs*. their respective areas. The curve was fitted to a logarithmic function [45]:



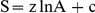



where S is the number of species, and A is the sampling area in cm^2^. To evaluate the model's performance, r^2^ was used as a standard goodness-of-fit measure. Based on this equation, the parameter k was calculated, which describes the shape of the curve and provides information on the qualitative distribution of species within the community [44], [46]:



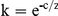



The higher the value of k, the larger the sampling area needed to obtain a representative number of species in the community due to their more dispersed distribution [44]. In this study, the qualitative minimal sampling area was determined as the point at which an increase of the sampling area by 20% yields a 5% increment in species number (Molinier point M 20/5) using the following equation:



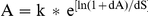



where dA and d'S are the relative increments of the surface area and species number (expressed as percentages), respectively. Hence, the Molinier point chosen in this study can be expressed as M 20/5 =  Amin  = 38.3 * k [44].

#### b) Estimation of sampling effort needed to maximize species number

In communities with a patchy distribution of species, such as coralligenous assemblages [8], combining small separate areas will usually result in a higher species count than will be obtained for a contiguous area of the same size [47]. Therefore, we also determined the minimal number of separate quadrats required to assess the maximum number of species present at each site (hereafter random quadrats). Consequently, we produced a second set of species-area curves based on 999 permutations, ignoring the spatial arrangement of these quadrats.

Finally, we also explored the increase in the number of species associated with increasing surface area when the MSAs determined for each assemblage were considered as sampling units (replicates).

### Tests for pattern assessment within the coralligenous outcrops

We applied multivariate analytical procedures to explore the suitability of the proposed methods for the detection of the variability of biodiversity within coralligenous outcrops on different spatial scales and among the two studied assemblages. More specifically, we explored whether the methods were able to cope with the intraregional variability (hundreds of meters to a few kilometers) and interregional variability (hundreds of kilometers) in the species composition of the two selected assemblages. Finally, we also explored the existence of differences between these assemblages.

Because many statistical analyses (e.g., analysis of variance) use replicate measurements to account for the amount of variation, we decided to use the MSA values obtained in this study (8 contiguous quadrats, see Results and Table 1) as replicates. Therefore, prior to analysis, presence/absence data were expressed for combinations of 8 contiguous quadrats ( =  replicates, measuring 50×100 cm for *PCA* and 40×80 cm for *CRA*). The total number of replicates per site ranged from 5 to 10.

**Table 1:** Logarithmic functions fitted to spatially explicit species-area curves based on the original order of contiguous samples.

Region	Site		Function	r^2^	k	A_min_/cm^2^
a) *Paramuricea clavata* assemblage						
Catalonia	El Medallot	(MME)	y = 9.26ln(x) - 45.09	0.99	131	4999
	El Tascó Petit	(MPT)	y = 6.84ln(x) - 27.16	0.973	53	2029
	Carall Bernat	(MRB)	y = 8.57ln(x) - 40.83	0.988	117	4481
Provence	Petit Conglué	(PCO)	y = 9.29ln(x) - 49.27	0.998	202	7718
	Plane-Grotte Pérès	(PGP)	y = 10.66ln(x) - 55.2	0.992	177	6787
Corsica	Gargallu	(SGL)	y = 8.68ln(x) - 41.59	0.996	121	4622
	Palazzino	(SPL)	y = 6.85ln(x) - 29.97	0.999	80	3050
	Palazzu	(SPA)	y = 9.04ln(x) - 43.57	0.995	124	4755
b) *Corallium rubrum* assemblage						
Catalonia	Cova de la Reina	(MRN)	y = 9.19ln(x) - 43.47	0.984	113	4336
	Cova de Dofí	(MGD)	y = 5.46ln(x) - 21.33	0.997	50	1899
Provence	Riou-Grotte Riou Sud	(RRS)	y = 5.49ln(x) - 20.39	0.987	41	1573
	Plane-Grotte Pérès	(PGP)	y = 5.89ln(x) - 19.67	0.969	28	1079
	Maïre Grotte à Corail	(MGC)	y = 5.83ln(x) - 22.92	0.999	51	1950
Corsica	Palazzu	(SPA)	y = 7.61ln(x) - 36.51	0.922	121	4645
	Passe Palazzu	(SPP)	y = 4.48ln(x) - 18.79	0.978	66	2530

Logarithmic functions, goodness of fit measure (r^2^), k parameter and minimal sampling areas (A_min_) calculated for each study site of the *Paramuricea clavata* and *Corallium rubrum* assemblages in the 3 regions of the NW Mediterranean. Site names are provided with abbreviations.

To determine the minimum number of replicates needed to assess biodiversity patterns, we compared the outcomes of the analysis using the overall dataset (all replicates available per site) and those using 3, 4, 5 and 6 replicates.

Similarly, we explored the potential effects on biodiversity patterns when smaller sampling unit sizes were used. For this purpose, we compared the results of a multivariate analysis based on a dataset using MSA values as replicates with those based on a dataset using single quadrats as replicates (25×25 cm for *PCA* and 20×20 cm for *CRA*).

### Data treatment

A Bray-Curtis similarity [48] matrix was constructed on the basis of presence/absence data. Non-metric multidimensional scaling (MDS) ordination [49] was performed to visualize patterns of community similarities.

Non-parametric analysis of variance PERMANOVA [50] was used to test for spatial variability. We applied a hierarchical design with 2 factors: Region (3 levels), as a random factor, and Site (8 and 7 levels for *PCA* and *CRA*, respectively), as a random factor nested in Region. Tests of significance were based on 9999 permutations of residuals under a reduced model [51], [52]. One-way PERMANOVA was applied to test for differences in species composition between the two assemblages (fixed factor). The test of significance was based on 9999 unrestricted permutations of raw data. All computations were performed using the PRIMER v6 software program with the PERMANOVA+ add-on package [53], [54].

## Results

### Categories identified

A total of 93 macrobenthic taxa were identified: 7 macroalgae, 1 protozoan, 39 sponges, 10 anthozoans, 1 hydrozoan, 5 polychaetes, 21 bryozoans and 9 tunicates (Table S1). Following appropriate grouping and elimination of seasonal taxa (see Methods), a total of 77 taxa were retained for further analysis. Of these, 75 taxa were recorded in *PCA* and 72 taxa in *CRA*. A total of 23 taxa were present in all regions within both communities, while 5 taxa were recorded exclusively within *PCA* and 2 taxa within *CRA* (Table S1). Of all identified categories (including taxa and groups), approximately 70 could be identified solely from photographs (without samples taken), upon a certain training. However, in general, the identification ability depended on the quality of photographs examined as well as whether the organisms were present in a typical morphological form or not (e.g., for the bryozoan *Turbicellepora* sp.).

### Determination of sampling method

#### Minimal sampling area (MSA)

**Figure 3:**
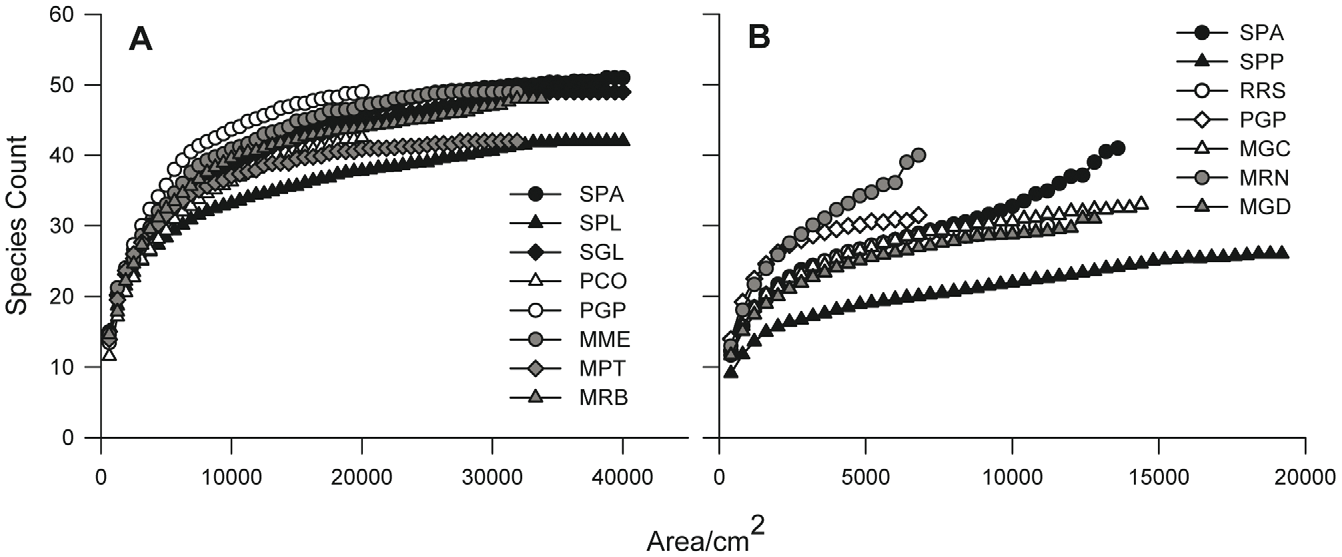
Spatially explicit species-area curves for each site within the 3 regions of the NW Mediterranean. (A) *Paramuricea clavata* assemblage and (B) *Corallium rubrum* assemblage (black  =  Corsica, white  =  Provence and gray  =  Catalonia). In a given area, each point represents multiple measures obtained from a subset of all possible combinations of increasing numbers of the originally ordered contiguous samples, with the curve based on the mean of those measures (SD not shown). See Table 1 for site abbreviations.

Spatially explicit species-area curves exhibited a fairly similar shape in the case of *PCA*, whereas they were more variable both in their shape and relative completeness in the case of *CRA* (Fig. 3). A good fit of the function to the data was indicated by r^2^ values higher than 0.90 in all cases (Table 1).

The mean value for the qualitative minimal sampling areas was approximately 5000 cm^2^ for *PCA* and half the size, 2500 cm^2^, for *CRA* (Table 1). Bearing in mind the size of the quadrats used in this study (see methods), approximately 8 contiguous quadrats (corresponding to surfaces of 50×100 cm for *PCA* and 40×80 cm for *CRA*) should be used to reach the MSAs for both assemblages as a replicate for biodiversity assessment studies.

Similar inter-site differences in MSAs were observed within each assemblage (Table 1). For *PCA*, the estimated area varied between 2000 and 8000 cm^2^, with the sites from the Provence region showing the largest MSA (around 7000 cm^2^). In the case of *CRA*, the values obtained were slightly lower, varying between 1000 and 5000 cm^2^ (Table 1).

#### Estimation of minimum sampling effort to maximize species number

**Figure 4:**
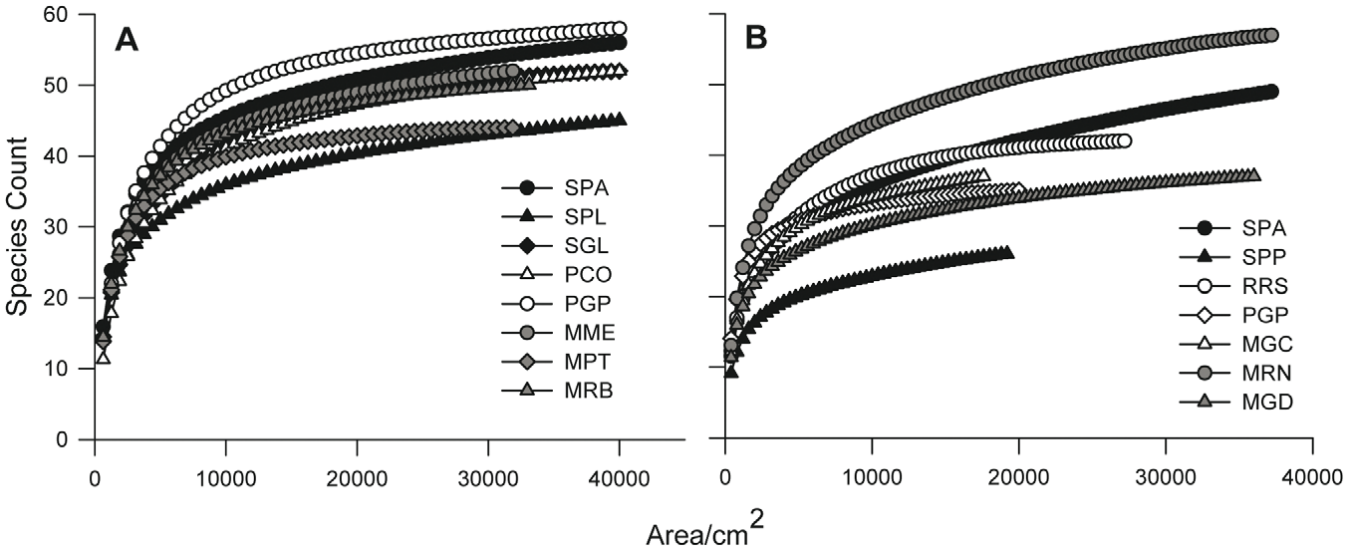
Spatially non-explicit species-area curves for each site within the 3 regions of the NW Mediterranean. (A) *Paramuricea clavata* assemblage and (B) *Corallium rubrum* assemblage (black  =  Corsica, white  =  Provence and gray  =  Catalonia). Data were based on 999 permutations of replicate samples (SD not shown). See Table 1 for site abbreviations.

Through analysis of all quadrats considered in this study, we determined the total number of species found at each site. For *PCA*, the species number ranged between 44 and 58, whereas for *CRA*, the number ranged between 26 and 57 (Table 2). Analysis of the species-area curves performed with random quadrats indicated that sampling efforts covering total areas of approximately 10,000 cm^2^ for *PCA* and 5000 cm^2^ for *CRA* would detect approximately 80% of all macrobenthic species recorded at the study sites (Fig. 4 and Table 2), whereas doubling the analyzed surface yielded more than 90% of the recorded species (Table 2). Therefore, to obtain good estimates of species number, approximately 16 to 32 random quadrats should be analyzed.

**Table 2:** The local species number per unit area estimated through spatially non-explicit species-area curves.

			Species				% Species			
Region	Sites	Total N	16	24	32	3×8	16	24	32	3×8
a) *Paramuricea clavata* assemblage										
Catalonia	El Medallot	52	44	47	49	44	84	90	94	85
	El Tascó Petit	44	40	42	43	40	91	95	97	91
	Carall Bernat	50	43	46	48	44	86	92	95	88
Provence	Petit Conglué	52	41	45	47	41	79	87	91	79
	Plane-Grotte Pérès	58	49	53	54	48	85	91	94	83
Corsica	Gargallu	52	41	45	48	40	80	87	92	77
	Palazzino	45	36	38	40	36	80	84	90	80
	Palazzu	56	45	49	51	45	81	88	91	80
b) *Corallium rubrum* assemblage										
Catalonia	Cova de la Reina	57	40	44	47	43	71	77	82	75
	Cova de Dofí	37	28	30	31	31	75	81	85	84
Provence	Riou-Grotte Riou Sud	42	33	37	39	36	80	88	92	86
	Plane-Grotte Pérès	35	32	33	34	32	90	94	97	91
	Maïre Grotte à Corail	37	32	34	35	34	85	92	95	92
Corsica	Palazzu	49	32	36	38	34	66	73	77	69
	Passe Palazzu	26	21	23	24	21	81	88	92	81

The local species number per unit area estimated through spatially non-explicit species-area curves (Fig. 4) for each site within each region. Total N: total number of species recorded at each site; Species: number of species observed by analyzing a different number of random quadrats (16, 24, 32) or a combination of contiguous quadrats (3×8 = 3 replicates of 8 contiguous quadrats); % Species: percentage of species observed in comparison to the total species number recorded. For random quadrats, calculations were based on 999 permutations of replicate samples, whereas for replicates of 8 contiguous quadrats, calculations were based on a subset of all potential replicate combinations (SD not shown).

When MSAs were used as sampling units, analysis of only 3 replicates of 8 contiguous quadrats provided approximately 80% of the total species found at each site (Table 2).

### Test for pattern assessment

#### a) Characterizing the regional variability of biodiversity patterns

**Figure 5:**
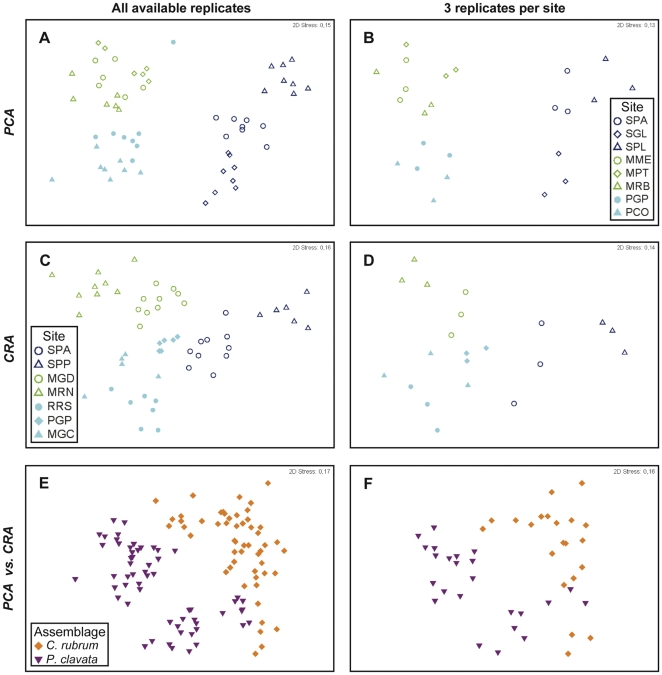
Non-metric multidimensional scaling (MDS) for all possible replicates and 3 replicates per site within the *Paramuricea clavata* (*PCA*) and *Corallium rubrum* (*CRA*) assemblages. Each replicate corresponds to 8 contiguous quadrats, creating a sampling unit of 50×100 cm for *PCA* and 40×80 cm for *CRA*. Three studied regions of the NW Mediterranean are depicted by colors (dark blue  =  Corsica, green  =  Catalonia and light blue  =  Provence). See Table 1 for site abbreviations.

Disregarding the number of replicates used per site (3, 4, 5 or 6), the patterns revealed by MDS and PERMANOVA were similar to those obtained using datasets based on the maximum possible number of replicates per site (5–10). Here, only the results of the analyses based on datasets with 3 and the maximum possible number of replicates per site (5–10) are shown (Fig. 5A–5D). For both assemblages, MDS ordination revealed 3 distinct clusters, corresponding to different regions (Fig. 5A and 5B; Fig. 5C and 5D), whereas PERMANOVA indicated significant variability at spatial levels for both region and site (Table 3). In the case of *PCA*, the greatest variation occurred at the regional scale, followed by sites and, finally, individual quadrats, whereas in the case of *CRA*, the greatest variation was observed at the site level, followed by regions and individual quadrats (Table 3). Similar levels of significance and explained variability were found, independent of the number of replicates used (Table 3).

**Table 3:** Summary of PERMANOVA analyses based on Bray-Curtis dissimilarity for macrobenthic taxa within the studied assemblages.

		A) *Paramuricea clavata* assemblage				B) *Corallium rubrum* assemblage			
Sampling unit and effort	Source	df	Pseudo-F	VC	BC diss (%)	df	Pseudo-F	VC	BC diss (%)
		AI) sampling unit size 50 cm×100 cm				BI) sampling unit size 40 cm×80 cm			
3 replicates	Region	2	56.19**	669.28	25.87	2	28.74*	408.70	20.22
	Site (Region)	5	40.83***	287.16	16.95	4	62.53***	418.79	20.46
	Residual	16		279.45	16.72	14		239.15	15.47
	Total	23				20			
4 replicates	Region	2	5.66**	625.67	25.01	2	2.52*	332	18.22
	Site (Region)	5	4.91***	280.34	16.74	4	9.54***	447.64	21.16
	Residual	24		287.08	16.94	21		209.57	14.48
	Total	31				27			
5 replicates	Region	2	6.17**	658.8	25.67	2	2.75**	363.79	19.07
	Site (Region)	5	6.17***	280.35	16.74	4	9.36***	424.56	20.61
	Residual	32		271.23	16.47	28		253.97	15.94
	Total	39				34			
6 replicates	Region	2	5.74**	632.5	25.15	2	2.64**	342.44	18.51
	Site (Region)	5	8.32***	308.11	17.55	4	11.14***	434.3	20.84
	Residual	40		252.65	15.90	34		249.31	15.79
	Total	47				40			
All replicates	Region	2	5.29**	607.82	25.00	2	2.33*	287.72	17.00
	Site (Region)	5	9.94***	331.93	18.00	4	13.76***	440.91	21.00
	Residual	50		267.3	16.00	46		249.28	16.00
	Total	57				52			
		AII) sampling unit size 25 cm×25 cm				BII) sampling unit size 20 cm×20 cm			
All quadrats	Region	2	2.68**	529.53	23.00	2	2.52*	396.64	20.00
	Site (Region)	5	37.32***	791.64	28.00	4	39.37***	548.78	23.00
	Residual	499		1367.3	37.00	479		932.88	31.00
	Total	506				485			

The results were obtained from datasets based on different number of replicates of 8 contiguous quadrats and individual quadrats. VC  =  Variance Components; BC diss  =  Bray Curtis dissimilarity.

P (perm) values.

*<0.05.

**<0.01.

***<0.001.

Likewise, the use of a different number of replicates did not change the outcome of comparisons of selected assemblages. In all cases, the MDS ordinations performed revealed two distinct clusters, clearly separating one assemblage from the other (Fig. 5E and 5F), while PERMANOVA indicated significant differences between them (Table 4).

**Table 4:** Summary of PERMANOVA analyses for the comparison of *Paramuricea clavata* (*PCA*) and *Corallium rubrum* (*CRA*) assemblages.

Sampling unit and effort	Source	df	Pseudo-F	VC	BC diss (%)
3 replicates	Assemblage	1	14.03***	558.22	23.63
	Residual	43		959.82	30.98
	Total	44			
All replicates	Assemblage	1	35.58***	561.93	23.71
	Res	109		899.97	30.00
	Total	110			
All quadrats	Assemblage	1	256.48***	1072.4	32.75
	Residual	959		2016.6	44.91
	Total	960			

The analyses were based on Bray-Curtis dissimilarity for macrobenthic taxa within the studied assemblages. The results were obtained from datasets based on different number of replicates of 8 contiguous quadrats and individual quadrats (25×25 cm for *PCA* and 20×20 cm for *CRA*). VC  =  Variance Components; BC diss  =  Bray Curtis dissimilarity.

P (perm) values:

*<0.05.

**<0.01.

***<0.001.

#### b) Analyzing the effect of different sampling unit sizes on biodiversity pattern assessment

The comparison of patterns using datasets based on individual quadrats (N = 475 for *PCA* and N = 486 for *CRA*) and 3 (or more) replicates of 8 contiguous quadrats revealed differences in the patterns and hierarchy of the spatial scales considered.

**Figure 6:**
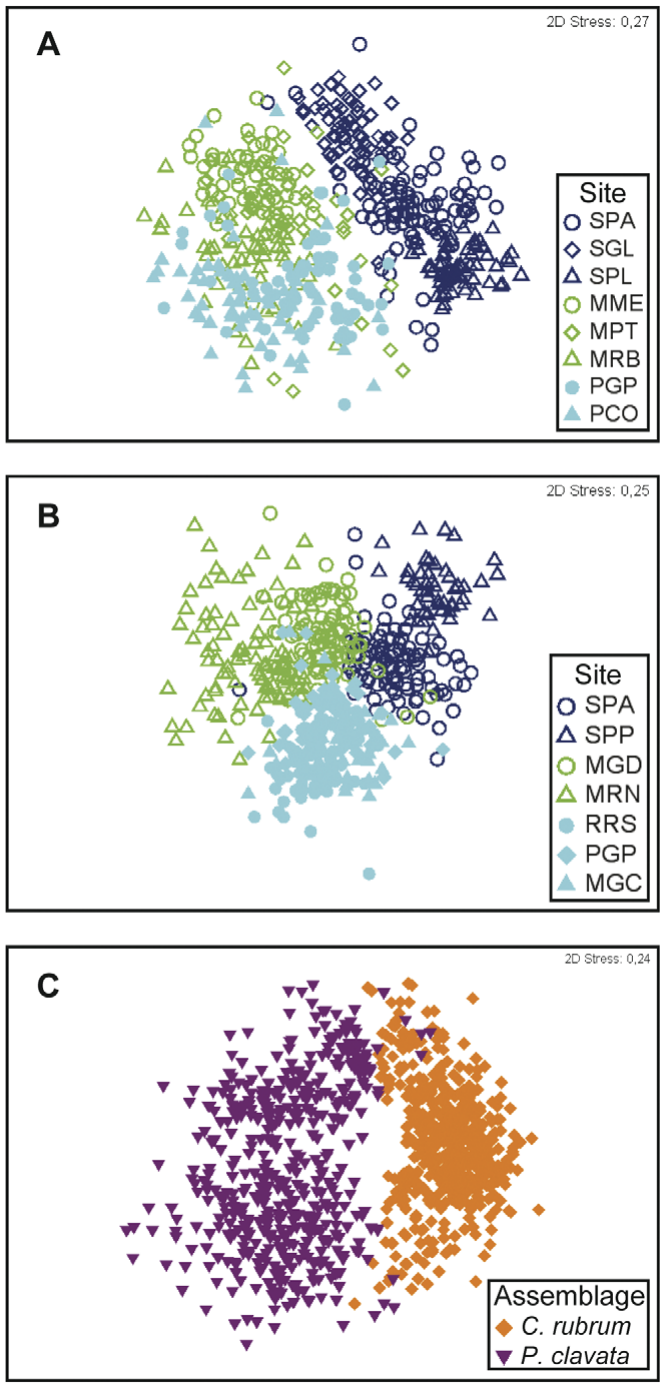
Non-metric multidimensional scaling (MDS) for the studied assemblages and their comparison. (A) *Paramuricea clavata* assemblage (sampling unit of 25×25 cm), (B) *Corallium rubrum* assemblage (sampling unit of 20×20 cm) and (C) comparison of *P. clavata* and *C. rubrum* assemblages in the 3 regions of the NW Mediterranean (dark blue  =  Corsica, green  =  Catalonia and light blue  =  Provence). See Table 1 for site abbreviations.

In the case of *PCA*, MDS ordination performed on the dataset based on individual quadrats revealed one distinct cluster corresponding to Corsica, whereas Catalonia and Provence overlapped (Fig. 6A). In the case of *CRA*, all clusters corresponding to different regions overlapped to a certain extent (Fig. 6B). In contrast, the MDS ordination performed on the dataset based on replicates of 8 contiguous quadrats clearly distinguished the regional clusters in both assemblages (Fig. 5A and 5C). While variability remained significant at both the region and site spatial levels, regardless of the dataset used, PERMANOVA revealed a different hierarchy of spatial scales depending on the sampling unit used. For both assemblages, in the case of datasets based on individual quadrats, the greatest component of variation was associated with the smallest spatial scale, i.e., individual quadrats (Table 3), whereas in the case of datasets based on replicates of 8 contiguous quadrats, the greatest component of variation was observed at larger spatial scales (regional level for *PCA* and site level for *CRA*). Finally, the use of smaller sampling units (individual quadrats) for comparison of selected assemblages revealed similar patterns to when larger sampling units (replicates of 8 contiguous quadrats) were used (Fig. 6C *vs*. Fig. 5E and 5F; Table 4), although the former method did not account for the particular structure of the assemblages because sampling unit size employed did not comply with the MSA.

## Discussion

Here, we propose, for the first time, a standardized biodiversity assessment method for coralligenous assemblages that provides good estimates of assemblage structure and species composition based on photographic sampling and determination of the presence/absence of macrobenthic species. We used an extensive photographic survey (almost 1000 photographs) covering several spatial scales (hundreds of meters to hundreds of kilometers) and including 2 different coralligenous assemblages (*PCA* and *CRA*), which allowed us to determine the MSA for each assemblage and optimize the sampling effort to assess biodiversity patterns and provide estimates of species number. Furthermore, we propose MSAs as unitary sampling units for variability studies requiring replicates. Three replicates measuring 5000 cm^2^ for *PCA* and 2500 cm^2^ for *CRA* were found to be sufficient to maximize the species number and to assess the main biodiversity patterns present (Tables 2 and 3). To ensure species identification and to facilitate the sampling procedures, we propose that photographs of smaller quadrats than the MSA arrayed to cover MSA surfaces should be obtained (e.g., 8 quadrats of 25×25 cm for *PCA* and 8 quadrats of 20×20 cm for *CRA*).

By combining a photographic survey and data acquired at the presence-absence level, the proposed method allows a large number of samples to be obtained during the limited diving time periods that are possible in deep water habitats (down to 50 m) [55], [56] and thus, to cope with the high spatial heterogeneity of coralligenous assemblages, while greatly reducing image time processing, which is one of the main constrains of photosampling. Recent studies comparing commonly used sampling methods in hard bottom communities also advocate the use of photo-quadrats attaining adequate sampling areas in change/impact studies or whenever a large number of replicates is needed [56], [57]. Additionally, the proposed protocol enables obtaining permanent objective records of both qualitative and quantitative data that can be further analyzed. For instance, analysis of species presence/absence datasets allows identifying the determinant species for such assemblages (SIMPER analysis, Primer, [58]), which can be further used to focus the quantitative (cover area) studies on these determinant species and thus optimize the image processing involved, alongside other methods that improve time efficiency in quantitative studies, such as recording frequencies instead of estimating cover [59] and/or applying an automated software [60]. Likewise, analysis of species presence/absence datasets a llows establishment of species area relationships (SARs), which have been recently proposed as indicators of community-level changes in biodiversity and may be useful in quantifying human impact [61].

One of the key aspects of the proposed method is the determination of MSAs as sampling units for the characterization of the coralligenous assemblages. To our knowledge, MSAs had only previously been estimated for studying cnidarian species dwelling in coralligenous assemblages [62], [63]. Interestingly, both studies determined comparable values for areas required to reach at least 80% of species: approximately 5000 cm^2^ for *PCA* and 4000 cm^2^ for *CRA*. In the present study, use of the MSA as a sampling unit was crucial for the assessment of biodiversity patterns. Comparison of the patterns obtained using MSA and smaller individual quadrats (used in the photo sampling) as replicates clearly showed a shift in the hierarchy of the estimates of variance components from large to small spatial scales. In general, the variation in the observed similarities among samples increases as the size of the sampling unit decreases [64]. Thus, using sampling units smaller than the MSA may have resulted in increased stochastic variability in the species composition at the smallest spatial scale. Similar effects have been reported previously in different habitats (e.g., [56], [65], [66]). However, previous studies on coralligenous outcrops adopted sampling units ranging between 240 and 600 cm^2^ (e.g., [21], [24]–[28], [67]–[69]), which were therefore much lower than MSA values, and found the highest variability at the replicate scale (e.g., [24], [25]). Hence, we emphasize the necessity to determine MSAs and use them as sampling units in the assessment of biodiversity patterns within coralligenous (and other) assemblages.

Although coralligenous assemblages harbor a significant proportion of the biodiversity that exists in the Mediterranean Sea [8], little is known about the biodiversity patterns within them. Bearing in mind the current pressures on coralligenous habitats [8], methods are urgently needed to assess prevailing patterns, evaluate impacts to which they are subjected and provide baseline data to explore future trajectories of these high diversity assemblages. We contend that the adoption of the method proposed in this study could furnish the required data to address these timely issues. In our opinion, three main research domains could be easily addressed using this method in a reasonable time framework to facilitate the development of meaningful management and conservation plans for coralligenous assemblages.

First, the method displayed potential for the characterization of biodiversity patterns. Its application to the analysis of spatial patterns at different scales (1 to 10^3^ km), including areas with differential environmental conditions and anthropogenic pressures, could help to establish conservation status baselines for coralligenous assemblages and, consequently, identify potential management actions needed for the recovery of areas with a low conservation status. Additionally, the method developed in this study could be used to address rarely surveyed deep coralligenous banks (extending from 60 down to 120 m, depending on the geographical position and local light conditions [8]), as ROVs (remotely operated vehicles) or research submersibles have the operational capability to collect high-resolution digital photographs that we contend are compatible with the proposed method. However, it has to be emphasized that the application of the proposed method for the assessment of deep coralligenous banks would be comparatively more difficult, since in our study scuba divers could manage to obtain the images even in coralligenous assemblages displaying high structural complexity (e.g. high density of vertical stratum) and/or developing on complex substrates such as overhangs or vaults. Obtaining the required sets of images with remote devices can be more challenging in deep coralligenous banks due to operational difficulties. Despite of this, we emphasize that the applicability of our approach is already suitable here by adapting the process of image acquisition. For instance, to ensure acquisition of spatially contiguous photographs of a standard size in these conditions of reduced operability at depth, individual still photographs could be obtained from a high resolution video transect. Besides, we strongly recommend to verify the actual number and size of replicates during the preliminary assessment, as the knowledge on the structure of deep coralligenous banks is very scarce. Finally, we believe that future technical advancements and improved operating abilities of ROVs/submersibles ensure the interest for developing biodiversity assessment methods based on the acquisition of images.

Second, the method could be applied to the evaluation of temporal changes in coralligenous assemblages, which would allow identification of impacts on the monitored assemblages. In this sense, it is crucial to establish temporal baselines to properly evaluate the significance of observed changes. Our results detected significant differences at the intra-regional scale, indicating that a reliable assessment of temporal trends should be carried out at the site level.

Finally, the proposed method proved to be sufficiently sensitive to detect significant differences between the studied coralligenous assemblages at both the community and geographic levels. Considering that coralligenous outcrops are regarded as a complex of assemblages [8], this approach may help to provide an objective basis to identify assemblages within coralligenous outcrops.

Application of unified sampling approaches over different regions, depths and times will allow tremendous progress to be made in our understanding of the biodiversity patterns of coralligenous outcrops. In this study, we developed a robust method for biodiversity assessment with the intention of providing a useful tool for management and conservation planning, monitoring and research programs focused on one of the most highly valued and emblematic Mediterranean habitats. We further contend that this method is potentially applicable in other benthic ecosystems.

## Supporting Information

Table S1 **List of the taxa identified in this study.** List of the taxa identified within the assemblages dominated by the red gorgonian *Paramuricea clavata* and the red coral *Corallium rubrum* in three regions of the NW Mediterranean.
(DOC)
