# Supplementary material for: Rapid Biodiversity Assessment and Monitoring Method for Highly Diverse Benthic Communities: A Case Study of Mediterranean Coralligenous Outcrops
Source: PLoS One. 2011 Nov 2;6(11):e27103. doi: 10.1371/journal.pone.0027103 (PMC3206946; doi:10.1371/journal.pone.0027103)
Supplement: Table S1 — List of the taxa identified in this study. List of the taxa identified within the assemblages dominated by the red gorgonian Paramuricea clavata and the red coral Corallium rubrum in three regions of the NW Mediterranean. (DOC) [file pone.0027103.s001.doc]

Table S1. **List of the taxa identified in this study.** List of the taxa identified within the assemblages dominated by the red gorgonian *Paramuricea clavata* and the red coral *Corallium rubrum* in three regions of the NW Mediterranean.

|  | *Paramuricea clavata* assemblage | | | *Corallium rubrum* assemblage | | |
| --- | --- | --- | --- | --- | --- | --- |
| **Taxa** | Catalonia | Provence | Corsica | Catalonia | Provence | Corsica |
| **Chlorophyta** | | | | | | |
| *Flabellia petiolata* | + | - | + | + | - | - |
| *Halimeda tuna* | - | - | + | - | - | + |
| *Palmophyllum crassum* | + | + | + | + | + | - |
| *Valonia macrophysa* | + | - | + | + | - | - |
| **Rhodophyta** | | | | | | |
| *Lithophyllum stictaeforme* | + | - | + | + | + | + |
| *Mesophyllum alternans* | + | + | + | + | + | + |
| *Peyssonnelia* sp. | + | + | + | + | - | - |
| **Protozoa** | | | | | | |
| *Miniacina miniacea* | - | + | + | + | + | + |
| **Porifera** |  |  |  |  |  |  |
| *Acanthella acuta* | + | + | + | + | + | + |
| *Agelas oroides* | + | + | + | + | + | + |
| *Aplysilla sulfurea^b^* | - | - | - | + | + | - |
| *Aplysina cavernicola* | - | + | + | + | + | - |
| *Axinella damicornis* | + | + | + | + | + | + |
| *Cacospongia* sp. | + | + | + | + | + | + |
| *Chondrosia reniformis* | + | + | + | + | + | - |
| *Clathrina clathrus* | + | + | + | + | - | + |
| *Clathrina coriacea~* | + | + | + | + | - | - |
| *Cliona* sp. | + | + | - | + | - | + |
| *Corticium candelabrum* | + | + | + | + | + | + |
| *Crambe crambe* | + | + | + | + | + | - |
| *Crella (Grayella) pulvinar* | + | + | + | + | + | + |
| *Dendroxea lenis* | + | + | - | + | + | + |
| *Dictyonella* sp. | + | + | + | + | + | - |
| *Fasciospongia cavernosa* | + | + | + | + | + | + |
| *Haliclona (Halichoclona) fulva* | + | - | + | + | + | + |
| *Haliclona (Reniera) mediterranea* | - | - | + | - | + | + |
| *Haliclona (Soestella) mucosa* | + | + | + | + | + | + |
| *Haliclona* sp. | - | + | + | + | - | + |
| *Hemimycale columella* | + | + | + | + | + | - |
| *Hexadella pruvoti* | + | + | - | + | - | + |
| *Hexadella racovitzai* | + | + | - | + | - | + |
| *Ircinia oros* | + | - | + | + | + | + |
| *Oscarella* sp. | + | + | - | + | + | + |
| *Petrosia ficiformis* | + | + | + | + | + | + |
| *Phorbas tenacior* | + | + | + | + | + | + |
| *Phorbas topsenti^b^* | - | - | - | - | + | - |
| *Plakina* sp. | - | - | + | + | + | + |
| *Plakortis* sp. | - | - | + | + | + | - |
| *Prosuberites longispinus* | + | + | + | + | + | + |
| *Raspaciona aculeata* | + | + | - | + | + | - |
| *Sarcotragus foetidus* | - | - | + | + | - | + |
| *Spirastrella cunctatrix* | - | + | + | + | + | + |
| *Spongia (Spongia) officinalis* | + | - | + | + | + | + |
| *Spongia virgultosa* | - | - | + | + | + | + |
| *Terpios granulosa* | + | + | + | + | + | - |
| Unidentified white calcareous sponge | + | + | + | - | + | - |
| Unidentified white Dendroceratida^^ | + | + | + | + | + | + |
| **Hydrozoa** | | | | | | |
| Unidentified Hydrozoa | + | + | + | + | + | + |
| **Anthozoa** | | | | | | |
| *Alcyonium acaule* | + | - | - | - | - | + |
| *Alcyonium coralloides* | + | + | + | - | + | - |
| *Caryophyllia inornata* | + | + | + | + | + | + |
| *Corallium rubrum* | - | + | - | + | + | + |
| *Corynactis viridis* | - | - | + | - | + | - |
| *Eunicella cavolini ^a^* | - | + | + | - | - | - |
| *Hoplangia durotrix* | + | + | + | + | + | + |
| *Leptopsammia pruvoti* | + | + | + | + | + | + |
| *Paramuricea clavata ^a^* | + | + | + | - | - | - |
| *Parazoanthus axinellae ^a^* | + | + | + | - | - | - |
| **Polychaeta** | | | | | | |
| *Filograna implexa / Salmacina dysteri* | + | + | + | + | + | + |
| *Protula* sp. | + | + | + | + | + | + |
| *Serpula vermicularis* | + | + | + | + | + | + |
| Serpulidae | + | + | + | + | + | + |
| **Bryozoa** | | | | | | |
| *Adeonella calveti*/*Smittina cervicornis* | + | + | + | + | - | + |
| *Beania hirtissima* | - | + | - | + | - | + |
| *Beania magellanica* | + | + | - | + | - | - |
| *Caberea boryi~* | + | + | + | - | + | + |
| *Chartella* sp. | + | - | + | - | - | + |
| *Cellaria* sp.~ | + | + | + | + | - | + |
| *Celleporina* sp.* | - | + | + | + | + | + |
| *Crisia* sp.~ | - | + | + | + | + | + |
| *Disporella hispida~* | + | - | + | + | + | + |
| *Dentiporella sardonica** | + | + | + | + | + | - |
| *Idmidronea* sp.~ | + | - | + | + | + | - |
| *Margaretta cereoides ^a^* | + | + | + | - | - | - |
| *Myriapora truncata* | + | + | + | + | - | - |
| *Pentapora fascialis ^a^* | - | + | + | - | - | - |
| *Reteporella grimaldii* | + | + | + | + | + | + |
| *Rhynchozoon* sp.* | + | - | - | + | - | + |
| *Schizomavella* sp.* | + | + | + | + | + | + |
| *Scrupocellaria* sp.~ | + | + | + | + | - | + |
| *Smittoidea* sp.* | - | - | + | + | + | + |
| *Turbicellepora* sp. | + | + | + | + | - | + |
| **Tunicata** | | | | | | |
| *Aplidium* sp. | - | + | + | + | + | + |
| *Aplidium undulatum* | - | - | + | + | + | + |
| *Clavelina dellavallei ^a^* ~ | - | - | + | - | - | - |
| *Clavelina lepadiformis ^a^* ~ | + | - | + | - | - | - |
| *Pycnoclavella nana* ~ | - | + | + | - | + | - |
| *Cystodytes* sp. | + | - | - | + | - | - |
| Unidentified Didemnidae | + | + | + | + | + | + |
| *Didemnum coriaceum* | - | + | - | + | - | + |
| *Halocynthia papillosa* | + | + | - | + | - | + |

* grouped as "encrusting bryozoans" in later analysis

~ species showing clear seasonality that were excluded from subsequent analysis

^^ includes the sponges *Pleraplysilla spinifera*, *Dysidea* sp. and *Aplysilla sulfurea*

*^a^* species found only in the *Paramuricea clavata* assemblage

*^b^* species found only in the *Corallium rubrum* assemblage
